# Vitamin K Dependent Protection of Renal Function in Multi-ethnic Population Studies

**DOI:** 10.1016/j.ebiom.2016.01.011

**Published:** 2016-01-13

**Authors:** Fang-Fei Wei, Nadja E.A. Drummen, Aletta E. Schutte, Lutgarde Thijs, Lotte Jacobs, Thibaut Petit, Wen-Yi Yang, Wayne Smith, Zhen-Yu Zhang, Yu-Mei Gu, Tatiana Kuznetsova, Peter Verhamme, Karel Allegaert, Rudolph Schutte, Evelyne Lerut, Pieter Evenepoel, Cees Vermeer, Jan A. Staessen

**Affiliations:** a Studies Coordinating Centre, Research Unit Hypertension and Cardiovascular Epidemiology, KU Leuven Department of Cardiovascular Sciences, University of Leuven, Belgium; b R & D Group VitaK, Maastricht University, Maastricht, The Netherlands; c Hypertension in Africa Research Team, North-West University, Potchefstroom, South Africa; d MRC Research Unit on Hypertension and Cardiovascular Disease, North-West University, Potchefstroom, South Africa; e Centre for Molecular and Vascular Biology, KU Leuven Department of Cardiovascular Sciences, University of Leuven, Belgium; f Research Unit Organ Systems, KU Leuven Department of Development and Regeneration, University of Leuven, Leuven, Belgium; g Department of Morphology and Molecular Pathology, University Hospitals Leuven, Leuven, Belgium; h Department of Nephrology and Renal Transplantation, University Hospitals Leuven, Leuven, Belgium

**Keywords:** Chronic kidney disease, Glomerular filtration rate, Matrix Gla protein, Population science, Vitamin K

## Abstract

**Background:**

Following activation by vitamin K (VK), matrix Gla protein (MGP) inhibits arterial calcification, but its role in preserving renal function remains unknown.

**Methods:**

In 1166 white Flemish (mean age, 38.2 years) and 714 South Africans (49.2% black; 40.6 years), we correlated estimated glomerular filtration (eGFR [CKD-EPI formula]) and stage of chronic kidney disease (CKD [KDOQI stages 2–3]) with inactive desphospho-uncarboxylated MGP (dp-ucMGP), using multivariable linear and logistic regression.

**Results:**

Among Flemish and white and black Africans, between-group differences in eGFR (90, 100 and 122 mL/min/1.73 m^2^), dp-ucMGP (3.7, 6.5 and 3.2 μg/L), and CKD prevalence (53.5, 28.7 and 10.5%) were significant, but associations of eGFR with dp-ucMGP did not differ among ethnicities (*P* ≥ 0.075). For a doubling of dp-ucMGP, eGFR decreased by 1.5 (*P* = 0.023), 1.0 (*P* = 0.56), 2.8 (*P* = 0.0012) and 2.1 (*P* < 0.0001) mL/min/1.73 m^2^ in Flemish, white Africans, black Africans and all participants combined; the odds ratios for moving up one CKD stage were 1.17 (*P* = 0.033), 1.03 (*P* = 0.87), 1.29 (*P* = 0.12) and 1.17 (*P* = 0.011), respectively.

**Interpretation:**

In the general population, eGFR decreases and CKD risk increases with higher dp-ucMGP, a marker of VK deficiency. These findings highlight the possibility that VK supplementation might promote renal health.

## Highlights

• In Flemish, eGFR was inversely associated with dp-ucMGP and positively with t-ucMGP.
• In Flemish, the risk of chronic kidney disease increased with higher dp-ucMGP.
• We confirmed the inverse association of eGFR with dp-ucMGP in black South Africans.

In a multi-ethnic population study, we assessed the association of renal glomerular filtration, a microvascular trait, with circulating matrix Gla protein (MGP). In white Flemish and in South African blacks, glomerular filtration decreased and the risk of chronic kidney disease increased with higher levels of inactive desphospho-uncarboxylated MGP. Our findings support the notion that following vitamin-K dependent carboxylation active MGP not only inhibits calcification of large arteries, as was known before, but also protects the renal microcirculation. Future studies might test whether vitamin K supplementation promotes renal health in the general population.

## 1 Introduction

Vascular smooth muscle cells synthesize matrix Gla protein (MGP), a small secretory protein (11 kD), which contains five γ-carboxyglutamate (Gla) amino-acid residues (Hackeng et al., 2001). Activation of MGP requires two posttranslational modifications: the vitamin-K dependent γ-glutamate carboxylation and serine phosphorylation (Schurgers et al., 2008). Carboxylated MGP is a potent inhibitor of arterial calcification (Schurgers et al., 2008). In patients with diabetes (Dalmeijer et al., 2013), renal dysfunction, (Schurgers et al., 2010) or macrovascular disease (Mayer et al., 2014), inactive desphospho-uncarboxylated MGP (dp-ucMGP) behaves as a circulating biomarker associated with cardiovascular risk (Dalmeijer et al., 2013), more severe vascular illness (Schurgers et al., 2010), and higher mortality (Mayer et al., 2014). In the Flemish Study on Environment, Genes and Health Outcomes (FLEMENGHO), circulating dp-ucMGP predicted total and cardiovascular mortality (Liu et al., 2015). Total uncarboxylated MGP (t-ucMGP), in contrast to dp-ucMGP, is not a marker of vitamin K status, but reflects arterial calcification, lower values being associated with more widespread calcium deposits (Cranenburg et al., 2009, Schurgers et al., 2005).

Previous research on MGP focused on macrovascular complications in patients (Dalmeijer et al., 2013, Schurgers et al., 2010, Mayer et al., 2014) or populations (Liu et al., 2015). MGP is abundantly expressed in the kidney with MGP immunoreactivity being associated with the epithelium of Bowman's capsule and the proximal tubules (Fraser and Price, 1988). Mineral nanoparticles containing calcium phosphate and calcification inhibitors are present in kidneys of patients with end-stage renal disease, but not healthy controls, and probably precede ectopic renal calcification (Wong et al., 2015). Moreover, calcification of the arterial wall is the hallmark of renal impairment (Lanzer et al., 2014, Moe and Chen, 2004) and may involve arterioles with a diameter as small as 10 to 500 μm (Lanzer et al., 2014). Based on these recent insights (Wong et al., 2015, Lanzer et al., 2014), we hypothesized that renal microvascular traits, such as glomerular filtration or microalbuminuria (Chade, 2013, Navar et al., 2008), might be adversely affected by deficient vitamin-K dependent activation of MGP, as exemplified by circulating dp-ucMGP. We investigated our hypothesis in white people enrolled in the FLEMENGHO study (Liu et al., 2015) and sought replication in the white and black participants enrolled in the South African Study Regarding the Influence of Sex, Age and Ethnicity on Insulin Sensitivity and Cardiovascular Function (SAfrEIC) (Kruger et al., 2012).

## 2 Methods

### 2.1 Recruitment of Participants

The two population studies complied with the Helsinki declaration for research in human subjects (World Medical Association, 2013), which were approved by the competent local ethics committees. Participants gave informed written consent. FLEMENGHO is a large-scale family-based population study, for which recruitment started in 1985 (participation rate, 78.0%) (Liu et al., 2015). The 3343 participants remained in follow-up, of whom 1179 had plasma dp-ucMGP and total uncarboxylated MGP (t-ucMGP) and serum and urinary creatinine and 24-h microalbuminuria measured at the same follow-up visit (Fig. S1). Black and white SAfrEIC residents of the Potchefstroom district in the North West Province of South Africa were recruited in 2007 (Kruger et al., 2012). Via advertisements or solicitation by community workers, apparently healthy people from 20 to 70 years old were invited (Kruger et al., 2012). Of 754 applicants complying with the entry criteria, 735 had both plasma dp-ucMGP and serum creatinine available for analysis. We excluded participants from analysis (Fig. S1), if they were taking warfarin (2 Flemish and 4 Africans), if MGP levels were more than 3 SDs away from the population mean (3 Flemish and 8 Africans), or if required covariables were unavailable (8 Flemish and 9 Africans). Thus, the number of participants statistically analyzed amounted to 1166 white Flemish and 714 South Africans, whose self-declared ethnicity was white in 362 and black in 352.

### 2.2 Clinical Measurements

In both the Flemish and African cohorts blood pressure was measured after participants had rested for at least 5 min in the seated position. In FLEMENGHO (Liu et al., 2015), blood pressure was the average of five consecutive auscultatory readings obtained with a standard mercury sphygmomanometer. In SAfrEIC (Schutte et al., 2010), blood pressure was the average of two oscillometric readings acquired by the OMRON HEM-757 device (El Assaad et al., 2003) (Omron Healthcare, Kyoto, Japan). Hypertension was a blood pressure of at least 140 mmHg systolic or 90 mmHg diastolic, or use of antihypertensive drugs. Trained nurses administered questionnaires inquiring into each participant's medical history, smoking and drinking habits, and intake of medications. Body mass index was weight in kilograms divided by height in meters squared.

### 2.3 Biochemical Measurements

Blood samples collected from an antecubital vein after the participants had been fasting for 6 to 8 h were analyzed for glucose and the serum levels of total and high-density lipoprotein (HDL) cholesterol, creatinine and γ-glutamyltransferase (biomarker of alcohol intake), using automated methods in certified laboratories. Diabetes mellitus was a fasting glucose exceeding 7.0 mmol/L (126 mg/dL) or use of antidiabetic agents (Expert Committee on the Diagnosis and Classification of Diabetes Mellitus, 2003). Estimated glomerular filtration rate (eGFR) was derived from serum creatinine, according to the Chronic Kidney Disease Epidemiology Collaboration (CKD-EPI) equation (Levey et al., 2009). We staged chronic kidney disease (CKD) according to the National Kidney Foundation (KDOQI) guideline (Levey et al., 2005) as eGFR ≥ 90, 89–60, 59–30 mL/min/1.73 m^2^.

Flemish participants collected a timed 24-h urine sample for the measurement of microalbumin and creatinine. Microalbuminuria was an albumin-to-creatinine ratio of at least 3.5 mg/mmol in women or 2.5 mg/mmol in men (Mancia et al., 2013). Human immunodeficiency virus (HIV) status was determined in South Africans with rapid tests according to the protocol of the National Department of Health (Pretoria) and confirmed with the Pareeshak test (BHAT Bio-tech, Bangalore, India) (Kruger et al., 2012).

For measurement of MGP, plasma was immediately separated from whole blood by 10 min of centrifugation at 1500 g-force. Plasma aliquots of 2 mL were deep frozen at − 20 °C or − 80 °C within 30 min of blood sampling. For long-term storage exceeding 2 months, all samples were kept at − 80 °C until assayed. dp-ucMGP (both cohorts) and t-ucMGP (Flemish only) were measured by pre-commercial ELISA kits at VitaK (Maastricht University, The Netherlands) (Cranenburg et al., 2010). The concentration of dp-ucMGP was assessed using the ina*K*tif MGP iSYS kit (Immunodiagnostic Systems Ltd., Boldon, UK), which is a dual-antibody test based on the sandwich ELISA developed by VitaK. Circulating t-ucMGP levels were measured by a competitive mono-antibody ELISA of the same origin.

### 2.4 Statistical Analyses

For database management and statistical analysis, we used the SAS system, version 9.4 (SAS Institute Inc., Cary, NC). Significance was a two-tailed α-level of 0.05 or less. Means and proportions were compared using the large-sample z-test or ANOVA and Fisher's exact test, respectively. We normalized the distributions of dp-ucMGP, t-ucMGP and γ-glutamyltransferase by a logarithmic transformation.

We determined differences in renal function across thirds of the MGP distributions from generalized linear models. In multivariable-adjusted linear and logistic regression with eGFR (continuous) or stage of chronic kidney disease (categorical) as outcome, we entered as covariables mean arterial pressure, heart rate, fasting glucose, the HDL-to-total cholesterol ratio, γ-glutamyltransferase, smoking, and treatment with antihypertensive drugs. Additional covariables in South Africans included HIV carrier state and in Flemish antihypertensive drug treatment, broken down into diuretics (thiazides, loop diuretics and aldosterone antagonists), β-blockers, inhibitors of the renin-angiotensin system (angiotensin-converting enzyme inhibitors and angiotensin type-1 receptor blockers), and vasodilators (calcium-channel blockers and α-blockers). For microalbuminuria, measured in Flemish only, we additionally adjusted models for sex, age, and body mass index. We expressed multivariable-adjusted association sizes between indexes of renal function and circulating MGP for a doubling of the biomarker. We determined differences in these associations between country of origin or ethnicity from the appropriate interaction terms. We also used multivariable mixed models, in which country, ethnicity or both were introduced as random effects and the other covariables as fixed effects.

## 3 Results

### 3.1 Characteristics of Participants

Table 1 provides the characteristics of participants by ethnic group. eGFR was significantly lower in white Flemish than in white South Africans and in white compared with black South Africans (*P* < 0.0001; Table 1). Indeed, eGFR averaged (SD) 89.5 (19.8) mL/min/1.73 m^2^ (range, 31.6–148.9) in Flemish, 100.3 (20.3) mL/min/1.73 m^2^ (range, 39.0–156.5) in white South Africans, and 121.8 (21.7) mL/min/1.73 m^2^ (range, 58.4–184.7) in black South Africans (Table 1). Circulating dp-ucMGP was lower in black South Africans than in white Flemish (*P* = 0.002), who in turn had lower dp-ucMGP levels than white South Africans (*P* < 0.0001). The geometric mean concentrations were 3.20 μg/L (range, 0.22–15.0) in black South Africans, 3.68 μg/L (0.23–27.8) in white Flemish, and 6.54 μg/L (0.56–25.6) in black South Africans. In Flemish, the geometric mean of t-ucMGP was 45.2 (range, 3.35–168.1) mg/L.

In Flemish, in whom blood samples were collected from 29 April 1996 until 29 November 2011 (5th to 95th percentile interval, 14 October 1996 until 18 July 2005), the MGP distributions did not materially differ according to the date of blood sampling (Fig. S2). Compared with Africans (*P* ≤ 0.0004), Flemish were younger (38.2 *vs.* 40.6 years) and had lower plasma glucose (5.02 *vs.* 5.34 mmol/L) and serum γ-glutamyltransferase (17.8 *vs.* 44.7 units/L). Compared with blacks (*P* ≤ 0.0007), whites had lower diastolic pressure (78.4 *vs.* 84.6 mmHg), lower heart rate (66.5 *vs.* 71.2 beats per minute) and a lower prevalence of hypertension (385 [25.2%] *vs.* 120 [34.1%]), whereas the opposite was true for treatment rates of hypertension (239 [62.3%] *vs.* 0 [0%]). Men compared with women were more likely to be smokers among Flemish (139 [24.5%] *vs*. 117 [19.6%]) and Africans (156 [48.0%] *vs*. 108 [27.8%]) or to report alcohol intake (433 [76.2%] *vs*. 287 [48.0%] and 257 [79.1%] *vs*. 221 [56.8%], respectively). The prevalence of HIV carrier state was 1 (0.3%) and 104 (29.6%) among white and black Africans.

In Flemish (Fig. S3), dp-ucMGP increased whereas eGFR decreased with age. Furthermore, in Flemish (Table S1), age, body mass index, systolic and diastolic blood pressure, prevalence of hypertension, blood glucose and serum total cholesterol and γ-glutamyltransferase increased (*P* ≤ 0.029) across thirds of the dp-ucMGP distributions, whereas HDL cholesterol and the HDL-to-total cholesterol ratio showed opposite trends (*P* ≤ 0.022). These observations were consistent among white and black Africans (Table S2). Measurements of t-ucMGP were available in Flemish only (Table S3). Body mass index, total cholesterol and γ-glutamyltransferase increased with higher t-ucMGP (*P* ≤ 0.0006), whereas the opposite was the case for HDL cholesterol and the HDL-to-total cholesterol ratio (*P* ≤ 0.0033).

### 3.2 Unadjusted Analyses of Renal Function

Among 1166 Flemish, 543 (46.6%) were in stage 1 of chronic kidney disease, 563 (48.3%) in stage 2, and 60 (5.2%) in stage 3; among 714 South Africans, these numbers were 573 (80.2%), 129 (18.1%), and 12 (1.7%), respectively (Table 2). Only 7 Flemish and 2 white Africans had an eGFR less than 45 mL/min/1.73 m^2^. No participant of either country had an eGFR below 30 mL/min/1.73 m^2^. Across increasing categories of dp-ucMGP (Table 2 and Fig. S4), eGFR decreased (*P* < 0.0001) and the prevalence of chronic kidney disease increased (*P* ≤ 0.0093). Among Flemish participants, microalbuminuria was present in 53 (4.6%), but was not associated with dp-ucMGP (Table 2; *P* ≥ 0.59). Neither eGFR nor microalbuminuria was associated with t-ucMGP (*P* ≥ 0.32; Table S4 and Fig. S4).

Unadjusted analyses of dp-ucMGP as continuous variable appear in Table S5. eGFR decreased (*P* < 0.0001) and the risk of renal dysfunction increased (*P* ≤ 0.024) with higher dp-ucMGP in white Flemish (n = 1166), black South Africans (n = 352), all South Africans (n = 714), and in all participants combined (n = 1880), whereas none of these associations was significant in white South Africans (n = 362; *P* ≥ 0.25).

### 3.3 Multivariable-Adjusted Analyses

**Fig. 1:**
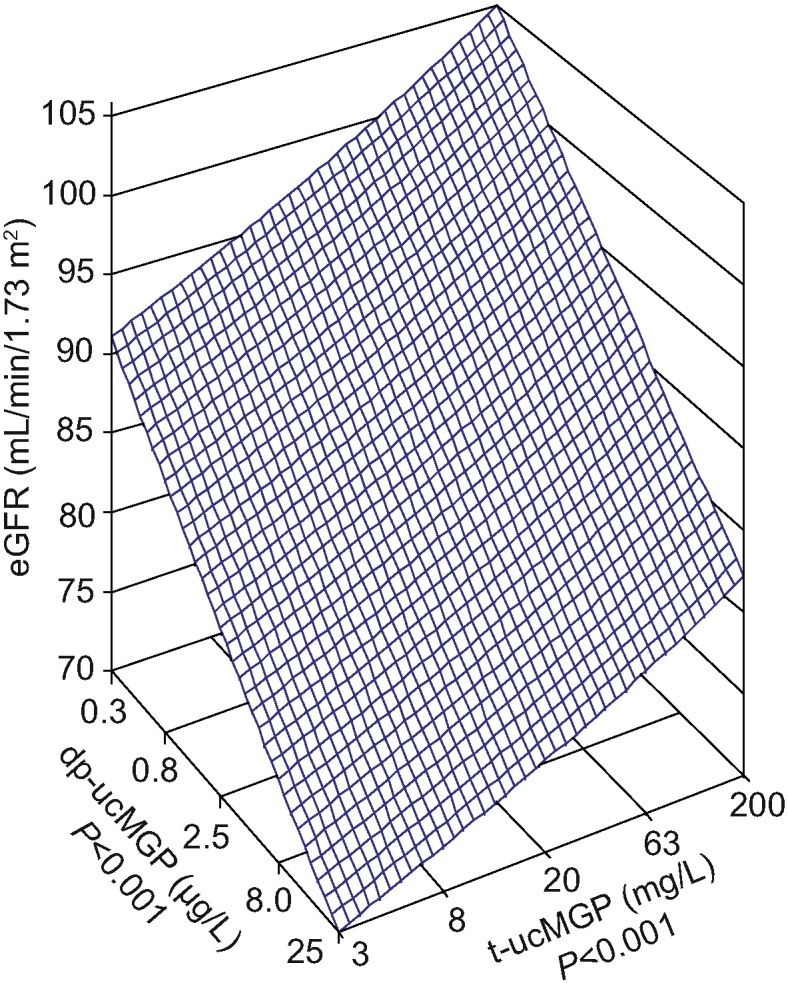
Multivariable-adjusted associations of estimated glomerular filtration rate with circulating matrix Gla proteins in Flemish participants. The plane shows the independent associations of eGFR (glomerular filtration rate derived from the serum creatinine concentration by Chronic Kidney Disease Epidemiology Collaboration equation) with dp-ucMGP (desphospho-uncarboxylated matrix Gla protein) and t-ucMGP (total uncarboxylated matrix Gla protein). The plotted plane was standardized to the mean distribution in the whole study population of mean arterial pressure, heart rate, HDL-to-total cholesterol ratio, plasma glucose, smoking, γ-glutamyltransferase, and treatment with diuretics, β- blockers, inhibitors of the renin-angiotensin system and vasodilators. dp-ucMGP increases with vitamin K deficiency, whereas t-ucMGP decreases with prevalent calcifications.

In multivariable-adjusted analyses with effect sizes expressed for a doubling of dp-ucMGP (Table 3), eGFR decreased by 1.46 mL/min/1.73 m^2^ (*P* = 0.023) in Flemish and by 2.78 mL/min/1.73 m^2^ (*P* = 0.0012) in black Africans with a directionally similar but substantially weaker association in white Africans (− 1.00 mL/min/1.73 m^2^; *P* = 0.56). These findings remained consistent if models for Flemish were adjusted for individual antihypertensive drug classes instead of all classes combined in a summary variable or if in blacks HIV carrier status was introduced as an additional covariable (Table 3). In Flemish, for a doubling of t-ucMGP, eGFR increased by 1.91 mL/min/1.73 m^2^ (95% confidence interval [CI], 0.08 to 3.73; *P* = 0.038). In models including both MGP conformations, the effect sizes were − 1.42 mL/min/1.73 m^2^ (CI, − 2.66 to − 0.19; *P* = 0.026) for dp-ucMGP and 1.86 (CI, 0.03 to 3.68; *P* = 0.044) for t-ucMGP (Fig. 1). In models additionally adjusted for country of origin, ethnicity or both, the association sizes of eGFR with dp-ucMGP were − 1.33 (*P* = 0.031) in all whites (n = 1528), − 2.55 (*P* = 0.0007) in Africans (n = 714), and − 2.07 (*P* < 0.0001) in all participants (n = 1880). Interactions of dp-ucMGP with country (*P* ≥ 0.39) or ethnicity (*P* ≥ 0.075) were not significant.

Table 3 lists the multivariable-adjusted odds ratios describing the risk of moving up one stage in the classification of chronic kidney disease associated with a doubling of dp-ucMGP. The odds ratios amounted to 1.17 (*P* = 0.033) in Flemish, and 1.29 (*P* = 0.12) and 1.03 (*P* = 0.87) in black and white South Africans. Alternatively adjusted models (Table 3) in Flemish and black Africans were confirmatory. The odds ratios in multivariable-adjusted logistic models that additionally include stratification variables were 1.15 (*P* = 0.049) in whites, 1.19 (*P* = 0.16) in Africans, and 1.17 (*P* = 0.011) in all participants (interaction *P*-values, ≥ 0.16). Mixed models with eGFR or stage of chronic kidney disease as dependent variable and including country of origin and ethnicity modeled as random effects and the other covariables as fixed effects also produced consistent results.

## 4 Discussion

We assessed the association of renal microvascular function as exemplified by eGFR with both circulating dp-ucMGP and t-ucMGP in a multi-ethnic population study. High dp-ucMGP is a marker of vitamin K deficiency (Dalmeijer et al., 2012), whereas t-ucMGP decreases with prevalent vascular calcification (Cranenburg et al., 2009, Schurgers et al., 2005). In Flemish, with adjustments applied for covariables, eGFR measured on a continuous scale was inversely associated with dp-ucMGP and positively with t-ucMGP. Furthermore, in categorical analyses, the risk of moving up one stage in the classification of chronic kidney disease increased with higher dp-ucMGP. We confirmed the inverse association of eGFR with dp-ucMGP in black South Africans and all South Africans combined. The potential relevance of these observations relates to the global epidemic of chronic kidney disease (Wang et al., 2012, Vos et al., 2012). The Global Burden of Disease Study 2010 collaboration estimated that worldwide 0.403 million of nearly 50 million deaths occurring annually, were attributable to renal failure in 1990 and 0.736 in 2010, representing an increase by 82.3% (Wang et al., 2012). Across all ages, over the same time span, the years lived with chronic kidney disease increased by 57.1% from 2.56 to 4.02 million, while the disability-adjusted life years, a metric that captures both premature mortality and the prevalence of ill-health caused by renal disease increased by 51.7% from 13.9 to 21.2 million (Vos et al., 2012).

Circulating levels of vitamin K are rarely measured in clinical practice, because of the complexity of the assay and the lack of a high-throughput method (Riphagen et al., 2015), and because plasma levels only reflect dietary intake (vitamin K_1_; phylloquinone) and production by the gut microflora (vitamin K_2_; menaquinones) over few hours without any indication of functionality (Dalmeijer et al., 2012, Westenfeld et al., 2012), whereas what really counts is the tissue level of active carboxylated MGP. We convincingly showed that high levels of dp-ucMGP indicate poor vitamin K status (Dalmeijer et al., 2012, Westenfeld et al., 2012). For instance, among 60 middle-aged healthy volunteers randomized in a placebo-controlled double-blind trial, circulating dp-ucMGP dropped dose-dependently by 31% and 46% in response to supplementation with 180 μg and 360 μg of menaquinone-7 (vitamin K_2_) daily for 12 weeks, whereas no changes occurred in t-ucMGP (*P* = 0.23) (Dalmeijer et al., 2012). Along similar lines, among 174 patients with chronic kidney disease, stages 3 to 5, the criteria for vitamin K deficiency were met in 6%, 60% and 97% based on plasma levels of phylloquinone, percent uncarboxylated osteocalcin, or proteins induced by vitamin K absence (PIVKA; des-γ-carboxy-prothrombin) (Holden et al., 2010). Poor vitamin K status in patients with chronic kidney disease or on hemodialysis not only results from higher needs of activation of MGP in the presence of the increased risk of arterial calcification, but also from lower dietary intake compared with healthy controls (Cranenburg et al., 2012). By far, the most extensive vascular calcifications occur in patients with chronic kidney disease (Lanzer et al., 2014, Moe and Chen, 2004). In keeping with our current findings, impaired inhibition of calcification might be a major player underlying the high risk of arterial calcification, macrovascular complications, and ultimately death in patients with chronic kidney disease (Moe and Chen, 2004). However, studies in selected patients with renal dysfunction cannot be generalized. In our Flemish population study, circulating dp-ucMGP measured at baseline, over 14.1 years of follow-up, predicted cardiovascular mortality and fatal combined with nonfatal cardiovascular events (Liu et al., 2015). Such events are caused by macrovascular disease. Our current cross-sectional studies in Flemish and South Africans, extend these observations to renal function, as exemplified by eGFR, a microcirculatory trait (Chade, 2013, Navar et al., 2008). In Flemish, in whom we measured the urinary excretion of microalbumin, the association with dp-ucMGP was not significant. However, in this Flemish population sample, the amount of microalbumin excreted and the prevalence of microalbuminuria (4.6%) were too low to reach statistical significance.

Both fetuin-A and MGP are inhibitors of calcification, but have complementary, albeit distinct, biological roles. Fetuin-A is a liver-secreted protein that is not vitamin K dependent and complexes with calcium and phosphate in the circulation to prevent precipitation of these minerals in tissues (Heiss et al., 2003, Schafer et al., 2003). In genetically engineered mice, knocking out MGP or fetuin-A results in arterial or soft tissue calcification or both (Schafer et al., 2003, Luo et al., 1997). Selectively reintroducing MGP expression in the liver of the MGP deficient mice, produced circulating MGP levels 6- to 10-fold higher than in wild type animals (Murshed et al., 2004). The MGP originating from the transgene conserved its biological activity in vitro, but did not inhibit arterial calcification (Murshed et al., 2004). Thus, fetuin-A is a systemically active protein, whereas MGP is locally synthesized and activated in vascular smooth muscle cells and prevents arterial calcification through local effects in the arterial wall. The recently introduced calcium propensity test (Pasch et al., 2012) measures a patient's systemic predisposition to extracellular matrix mineralization, which is controlled by fetuin-A, but probably not by locally synthesized MGP, as no correlation exits between circulating levels of fetuin-A and MGP (Javadzadeh et al., 2015).

In keeping with a previous report in 842 out-patients with stable cardiovascular disease (Parker et al., 2009), t-ucMGP correlated positively with eGFR. t-ucMGP, in the literature often referred to as ucMGP, predominantly consists of phosphorylated MGP and at variance with dp-ucMGP is not a biomarker of vitamin K status, but lower levels reflect more extensive cardiovascular calcifications (Cranenburg et al., 2009, Schurgers et al., 2005). t-ucMGP accumulates at arterial calcification sites, possibly by binding through its negatively charged phosphoserine residues (Cranenburg et al., 2009, Schurgers et al., 2005). Upregulation of MGP transcription in response to vascular stress influences circulating t-ucMGP levels (Cranenburg et al., 2009). In 40 patients on hemodialysis, the mean t-ucMGP level was significantly lower than in healthy age-matched controls. Additionally, higher coronary calcification scores determined by multi-slice computed tomography were associated with lower t-ucMGP levels (r = − 0.41; *P* = 0.009). This correlation persisted after adjustment for age, dialysis vintage, and high-sensitivity C-reactive protein as marker of inflammation (Cranenburg et al., 2009).

Several points related to the ethnic and lifestyle differences in our study deserve to be specifically highlighted. First, Flemish were randomly recruited from the general population. South Africans were also enrolled from the local community via advertisements and by invitation by health workers, but only qualified for participation if they were healthy. This difference in recruitment strategy explains the lower prevalence of hypertension and higher eGFR among South African compared with Flemish whites. Second, compared with whites, blacks have a higher glomerular filtration rate indicative of glomerular hyperfiltration (Kotchen et al., 2000). eGFR as estimated from serum creatinine by the CKD-EPI equation (Levey et al., 2009) accounts for this well established ethnic difference. In addition, blacks compared with whites are more susceptible to hypertension and its complications (Burt et al., 1995, Hara et al., 2015). The observation of a higher eGFR (Kotchen et al., 2000) and higher prevalence of hypertension among blacks (Burt et al., 1995, Hara et al., 2015) compared with whites is therefore in keeping with the literature and constitutes an external validation of our study. Finally, the association between eGFR and dp-ucMGP in white South Africans, although directionally consistent with the association in white Flemish and in black South Africans, did not reach significance. However, dp-ucMGP levels in white South Africans were approximately twice as high compared with white Flemish and their black counterparts. Elevated dp-ucMGP levels with little interindividual variation in white South Africans in the presence of normal variation in eGFR may have weakened the association (Fig. S5). Low dp-ucMGP reflects high intake of vitamin K, mostly vitamin K_2_. Fermented products are the main dietary source of menaquinones. Europeans get most of their intake from cheese. In South Africa, cheese consumption is much lower than in Europe. The per capita consumption is approximately 0.9 kg per year compared to 19.2 kg per year in Belgium (data for 2011 available at http://www.helgilibrary.com/indicators/index/cheese-consumption-per-capita). Traditionally, consumption of fermented foods is high in black populations of sub-Saharan Africa, particularly in rural areas, and makes up for the cheese as source of vitamin K in Flemish. Moreover, abuse of antibiotics is very common in South Africa (Gelband and Duse, 2011), in particular among affluent whites, which might adversely affect production of vitamin K_2_ by the gut microflora. In 2000, the per capita use of antibiotics was approximately 20 standard units in Belgium and 15 standard units in South Africa, all ethnicities combined (Gelband et al., 2015). From 2000 to 2010, the per capita use decreased by 18% in Belgium (Gelband et al., 2015) as a consequence of policies instituted by the Federal Government (Goossens, 2000), but over the same time span rose by 280% in South Africa (Gelband et al., 2015).

The current study must be interpreted within the context of its strengths and potential limitations. A strong point is that we measured both dp-ucMGP and t-ucMGP in a relatively large sample representative of the Flemish population and that we replicated the inverse association of eGFR or chronic kidney disease with dp-ucMGP in a multi-ethnic South African cohort. We demonstrated that our MGP assay was not sensitive to long-term storage of blood samples (Fig. S2). Generation of creatinine differs among ethnic groups (Stevens et al., 2011, Udler et al., 2015) and HIV carrier state affects estimates of eGFR and chronic kidney disease (Seape et al., 2016). However, among the creatinine-based equations, the CKD-EPI approach (Levey et al., 2009) performed best in estimating renal function in treatment-naïve HIV patients (Seape et al., 2016) and South African blacks (Matsha et al., 2013), and in predicting risk in over one million people recruited from 40 multi-ethnic cohort studies (Matsushita et al., 2012).

Among the potential limitations of our current study is its cross-sectional design, which precludes direct causal inference. Second, the historical record of migration into South Africa implies that genetic admixture is common place (Stull et al., 2014). Ethnicity among our South African participants was self-reported. However, for research purposes, experts still assign great value to racial self-categorization (Risch et al., 2002) and South African studies demonstrated good agreement between racial self-categorization and forensic (Stull et al., 2014) and genetic (Patterson et al., 2010) markers of race. Third, because of admixture and diversity in lifestyle and living environment, whites of Flemish and South African origin must be more dissimilar than just suggested by ethnicity. Finally, we could not reliably assess the dietary intake of vitamin K, because validated food frequency questionnaires and food composition tables are unavailable for use in Flemish or South African populations.

Notwithstanding potential limitations, our findings may have important clinical implications. High levels of plasma dp-ucMGP are a proxy for vitamin K deficiency (Dalmeijer et al., 2012, Westenfeld et al., 2012). Levels ranging from ~ 1.4 to ~ 4.6 μg/L are probably optimal in terms of the risk of mortality and macrovascular cardiovascular complications (Liu et al., 2015). In Flemish, the 4.6 μg/L threshold corresponded with the 65th percentile of the dp-ucMGP distribution, indicating that 35% of Flemish might be vitamin K deficient. In South Africa, lactic acid bacteria dominate the microflora of fermented milk products (Beukes et al., 2001), but with urbanization and the associated socioeconomic and lifestyle changes, traditional technologies for the production of fermented foods will eventually be lost together with the associated micro-organisms, thereby increasing the risk of vitamin K deficiency (Anukam and Reid, 2009). The recommended dietary allowance for vitamin K is 1 μg per kilogram of body weight per day, which is sufficient to ensure normal hemostasis (Weber, 2001). However, in apparently healthy individuals, a substantial fraction of MGP remains in the uncarboxylated forms (Schurgers et al., 2005). This raises the question as to whether the present recommended dietary allowance for vitamin K intake is sufficient to prevent macrovascular disease. In Swiss randomly recruited from the general population, aortic pulse wave velocity was positively associated with plasma dp-ucMGP, even after adjustment for cardiovascular risk factors and renal dysfunction (Pivin et al., 2015). Vitamin K supplementation reduced aortic pulse wave velocity in healthy postmenopausal women (Knapen et al., 2015). Assuming reversibility, our current findings extend the protective role of vitamin K from the macrocirculation to renal function and possibly reveal a potential for prevention by supplementation, for instance by biologically enriched fermented vegetable or dairy products. Moreover, vitamin K has a very wide safety range, irrespective whether the sources are leafy vegetables (phylloquinone; vitamin K_1_) or fermented foods (menaquinones; vitamin K_2_) (Weber, 2001). While dp-ucMGP might be a biomarker relevant to prevent disease, circulating t-ucMGP might find its way to clinical application as a marker of arterial calcification at a stage when macrovascular disease is still asymptomatic.

## 5 Conclusion

In white Flemish and black South Africans recruited from the general population, eGFR decreased and the risk of renal impairment increased with higher dp-ucMGP, a marker of vitamin K deficiency. These epidemiological findings support the concept that active MGP might not only inhibit calcification in large arteries, as was well known before (Knapen et al., 2015), but might also protect renal function. Our observations potentially highlight new avenues for promoting renal health, for instance by increasing the dietary intake of vitamin K either by supplementation or by increasing the intake of nutrients rich in vitamin K.

## Funding

The European Union (HEALTH-2011.2.4.2-2-EU-MASCARA, HEALTH-F7-305507 HOMAGE and the European Research Council Advanced Researcher Grant-2011-294713-EPLORE) and the Fonds voor Wetenschappelijk Onderzoek Vlaanderen, Ministry of the Flemish community, Brussels, Belgium (G.0881.13, G.088013 and 11Z0916N) currently support the Studies Coordinating Centre in Leuven. Furthermore, the South African Study received funding from the South African National Research Foundation (GUN 2073040), the Medical Research Council of South Africa, and the Africa Unit for Transdisciplinary Health Research (AUTHeR) of the North-West University (Potchefstroom Campus).

## Role of sponsors

The sponsors had no role in the design of the study, the collection and analysis of the data, or the preparation of the manuscript.

## Author Contributions

J.A. Staessen conceived and coordinated the Flemish Study on Environment, Genes and Health Outcomes (FLEMENGHO). L Thijs, L Jacobs, T Kuznetsova, and JA Staessen constructed the FLEMENGHO database. F-F Wei, T Petit, W-Y Yang, Z-Y Zhang and Y-M Gu did field work in Flanders. AE Schutte, R Schutte and W Smith provided the South African data. N Drummen and C Vermeer supervised the measurements of matrix Gla protein. K Allegaert, P Evenepoel, E Lerut and P Verhamme advised on interpretation of the microcirculatory and renal phenotypes. F-F Wei, C Vermeer, and JA Staessen wrote the first draft of the manuscript. All authors interpreted the results, commented on successive drafts of the manuscript, and approved the final version.

## Conflict of Interest

NAE Drummen and C Vermeer are employees of the VitaK R&D Group. The other authors have no conflict of interest.

## Figures and Tables

**Table 1:** Characteristics of participants.

Characteristic	White Flemish	White South Africans	Black South Africans	*P*
Number of participants (%)				
All participants in category	1166	362	352	
Women	598 (51.3)	205 (56.6)	184 (52.3)	0.20
Smokers	256 (22.0)	53 (14.6)†	211 (59.9)§	< 0.0001
Drinking alcohol	720 (61.8)	238 (65.8)	240 (68.2)	0.060
Hypertension	337 (28.9)	48 (13.3)§	120 (34.1)§	< 0.0001
Antihypertensive treatment	168 (14.4)	71 (19.6)*	0 (0)§	< 0.0001
Diabetes mellitus	53 (4.6)	27 (7.5)*	10 (2.8)†	0.013
Previous cardiovascular disease	35 (3.0)	3 (0.8)§	2 (0.6)	< 0.0001
Mean (SD) of characteristic				
Age (years)	38.2 (15.8)	40.3 (12.9)*	40.9 (11.5)	0.0018
Body mass index (kg/m^2^)	25.4 (4.3)	27.7 (5.8)§	24.0 (6.9)§	< 0.0001
Systolic pressure (mm Hg)	125.9 (14.8)	119.1 (15.9)§	125.4 (20.7)§	< 0.0001
Diastolic pressure (mm Hg)	78.5 (10.2)	78.1 (9.8)	84.6 (13.3)§	< 0.0001
Heart rate (beats per minute)	66.2 (9.3)	67.5 (9.2)*	71.2 (13.4)§	< 0.0001
Serum total cholesterol (mmol/L)	5.14 (1.05)	5.86 (1.42)§	4.36 (1.08)§	< 0.0001
Serum HDL cholesterol (mmol/L)	1.43 (0.40)	1.38 (0.41)	1.49 (0.63)†	0.0073
HDL-to-total cholesterol ratio	0.29 (0.09)	0.24 (0.08)§	0.35 (0.12)§	< 0.0001
Plasma glucose (mmol/L)	5.02 (1.22)	5.54 (1.19)§	5.13 (1.03)§	< 0.0001
Serum creatinine (μmol/L)	85.6 (14.8)	72.8 (12.7)§	67.4 (12.7)§	< 0.0001
eGFR (mL/min/1.73 m^2^)	89.5 (19.8)	100.3 (20.3)§	121.8 (21.7)§	< 0.0001
Geometric mean (IQR) of characteristic				
γ-glutamyltransferase (units/L)	17.8 (12.0–24.0)	30.5 (19.0–41.7)§	66.1 (30.5–125.9)§	< 0.0001
dp-ucMGP (μg/L)	3.68 (2.60–5.31)	6.54 (5.31–8.82)§	3.20 (2.07–6.54)§	< 0.0001

Abbreviations: dp-ucMGP, desphospho-uncarboxylated matrix Gla protein; IQR, interquartile range; HDL, high-density lipoprotein. To convert dp-ucMGP from μg/L into pmol/L, multiply by 94.299. Hypertension was a blood pressure of ≥ 140 mmHg systolic or ≥ 90 mmHg diastolic, or use of antihypertensive drugs. Diabetes mellitus was a fasting blood glucose ≥ 7.0 mmol/L (126 mg/dL) or use of antidiabetic agents. *P* values denote the significance of the overall difference in prevalence or mean between groups. Significance of the difference with the left adjacent group: * *P* ≤ 0.05; † *P* ≤ 0.01; ‡ *P* ≤ 0.001; § *P* ≤ 0.0001.

**Table 2:** Renal function by cohort and thirds of the dp-ucMGP distribution.

Characteristic	Category of dp-ucMGP				*P*
	**FLEMENGHO**				
Limits, (μg/L)	< 3.02	3.02–4.75	≥ 4.75		
Number of participants (%)					
All participants in category	388	389	389		
Microalbuminuria	19 (4.9)	13 (3.3)	21 (5.4)		0.36
Stage of chronic kidney disease					
1	200 (51.6)	196 (50.4)	147 (37.8)†		< 0.0001
2	184 (47.4)	176 (45.2)	203 (52.2)
3	4 (1.0)	17 (4.4)†	39 (10.0)†
Mean (SD) of characteristic					
Serum creatinine (μmol/L)	84.8 (12.6)	85.2 (14.7)	86.7 (16.8)		0.19
eGFR (mL/min/1.73 m^2^)	92.7 (17.6)	90.9 (19.8)	85.0 (21.1)§		< 0.0001
Geometric mean (IQR) of characteristic					
Urinary ACR (mg/mmol)	0.79 (0.45–1.35)	0.78 (0.41–1.44)	0.81 (0.45–1.38)		0.73
24-h microalbuminuria (mg)	8.5 (5.0–15.1)	8.1 (4.5–15.1)	8.5 (4.9–15.1)		0.59
	**SAfrEIC**				
Limits, (μg/L)	< 4.39	4.39–7.20	≥ 7.20		
Number of participants (%)					
All participants in category	237	238	239		
Stage of chronic kidney disease					
1	207 (87.3)	187 (78.6)	179 (74.9)		0.0093
2	28 (11.8)	45 (18.9)	56 (23.4)
3	2 (0.8)	6 (2.5)	4 (1.7)
Mean (SD) of characteristic					
Serum creatinine (μmol/L)	68.1 (12.4)	71.3 (13.2)†	71.0 (13.0)		0.014
eGFR (mL/min/1.73 m^2^)	121.2 (23.3)	108.1 (23.2)§	103.5 (20.6)*		< 0.0001

Abbreviations: dp-ucMGP, desphospho-uncarboxylated matrix GLA protein; eGFR, estimated glomerular filtration rate according to the Chronic Kidney Disease Epidemiology Collaboration (CKD-EPI) equation; IQR, interquartile range; ACR, urinary albumin-to-creatinine ratio. Microalbuminuria was an albumin-to-creatinine ratio ≥ 3.5 mg/mmol in women and ≥ 2.5 mg/mmol in men. Chronic kidney disease was staged according to the National Kidney Foundation (KDOQI) guideline as eGFR ≥ 90, 60–89, 30–59 mL/min/1.73 m^2^. *P* values denote the significance of the difference in prevalence or mean across thirds of the distribution of dp-ucMGP. Significance of the difference with the adjacent lower third: * *P* ≤ 0.05; † *P* ≤ 0.01; ‡ *P* ≤ 0.001; and § *P* ≤ 0.0001.

**Table 3:** Adjusted associations of renal function with desphospho-uncarboxylated matrix Gla protein.

Participants model	eGFR (mL/min/1.73 m^2^)		Chronic kidney disease	
Association size (95% CI)	*P*	Odds ratio (95% CI)	*P*
White Flemish				
Standard	− 1.46 (− 2.71 to − 0.20)	0.0230	1.17 (1.01 to 1.36)	0.033
Alternative	− 1.57 (− 2.83 to − 0.31)	0.0146	1.19 (1.02 to 1.38)	0.022
White South Africans	− 1.00 (− 4.42 to 2.42)	0.56	1.03 (0.70 to 1.53)	0.87
Black South Africans				
Standard	− 2.78 (− 4.45 to − 1.11)	0.0012	1.29 (0.94 to 1.79)	0.12
Alternative	− 2.80 (− 4.47 to − 1.13)	0.0011	1.30 (0.94 to 1.80)	0.12
All Whites	− 1.33 (− 2.52 to − 0.12)	0.0314	1.15 (1.00 to 1.31)	0.049
All South Africans	− 2.55 (− 4.02 to 1.08)	0.0007	1.19 (0.94 to 1.52)	0.16
All participants	− 2.07 (− 3.02 to − 1.12)	< 0.0001	1.17 (1.04 to 1.33)	0.011

Association sizes and odds ratios express the change in the dependent variable associated with a doubling of desphospho-uncarboxylated matrix Gla protein (dp-ucMGP). Standard models accounted for mean arterial pressure, heart rate, plasma glucose, HDL-to-total cholesterol ratio, γ-glutamyltransferase, smoking, and antihypertensive drug treatment. The alternative model in FLEMENGHO participants was adjusted for treatment with diuretics, β- blockers, inhibitors of the renin-angiotensin system, and vasodilators instead of all antihypertensive drug classes combined in a single variable. The alternative model in blacks was additionally adjusted for HIV carrier state. Covariables coding for the strata were entered into models including participants from two countries or two ethnicities. Interactions of dp-ucMGP with country of origin or ethnicity were not significant (*P* ≥ 0.075).
