# Supplementary material for: Vitamin K Dependent Protection of Renal Function in Multi-ethnic Population Studies
Source: eBioMedicine. 2016 Jan 13;4:162–9. doi: 10.1016/j.ebiom.2016.01.011 (PMC4776057; doi:10.1016/j.ebiom.2016.01.011)
Supplement: Supplementary file 1 — Supplementary material. [file mmc1.pdf]

EBioMedicine

Appendix A Supplementary Data

This appendix formed part of the original submission and has been peer reviewed.
Supplement to: "*Vitamin K Dependent Protection of Renal Function in Multi-Ethnic Population Studies"*.

Contents

Table S1. Characteristics of participants by cohort and thirds of the dp-ucMGP distribution.→→p2

Table S2. Characteristics of South African participants by ethnicity and thirds of the dp-ucMGP distribution.→→p3

Table S3. Characteristics of Flemish participants by thirds of the t-ucMGP distribution.→→p4

Table S4. Renal function in Flemish participants by thirds of the t-ucMGP distribution.→→p5

Table S5. Unadjusted associations of renal function with desphospho-uncarboxylated matrix Gla protein.→→p6

Fig. S1. Flow chart of participants.→→p7

Fig. S2. Boxplots of the distributions of matrix Gla protein by date of blood sampling in FLEMENGHO participants.→→p8

Fig. S3. Age dependency of circulating matrix Gla protein and estimated glomerular filtration rate in 1166 FLEMENGHO participants.→→p9

Fig. S4. Association between circulating matrix Gla protein levels and stage of chronic kidney disease in 1166 FLEMENGHO participants.→→p10

Fig. S5. Association between estimated glomerular filtration rate and desphospho-uncarboxylated matric Gla protein in 1880 participants.→→p11

Table S1

Characteristics of participants by cohort and thirds of the dp-ucMGP distribution.

Characteristic	FLEMENGHO			SAfrEIC		
	Category of dp-ucMGP			Category of dp-ucMGP		
Limits, (μg/L)	<3.02	3.02–4.75	≥4.75	<4.39	4.39–7.20	≥7.20
N° participants (%)						
N° in category	388	389	389	237	238	239
Women	192 (49.5)	192 (49.4)	214 (55.0)	108 (45.6)	139 (58.4)†	142 (59.4)
Smokers	106 (27.3)	96 (24.7)	54 (13.9)§	139 (58.6)	68 (28.6)§	57 (23.8)
Drinking alcohol	262 (67.5)	234 (60.2)*	224 (57.6)	162 (68.4)	166 (69.8)	150 (62.8)
Hypertension	71 (18.3)	111 (28.5)‡	155 (39.8)‡	64 (27.0)	42 (17.6)*	62 (25.9)*
Antihypertensive treatment	27 (7.0)	55 (14.1)†	86 (21.1)†	5 (2.1)	27 (11.3)‡	39 (16.3)
Diabetes mellitus	11 (2.8)	24 (6.2)*	18 (4.6)	5 (2.1)	11 (4.6)	21 (8.8)
Previous cardiovascular disease	6 (1.6)	11 (2.8)	18 (4.6)	1 (0.4)	2 (0.8)	2 (0.8)
Mean (SD)						
Age (years)	34.0 (12.6)	36.9 (14.8)†	43.6 (18.1)§	37.9 (11.3)	40.0 (12.6)	43.9 (12.2)‡
Body mass index (kg/m2)	24.4 (3.6)	25.1 (4.4)*	26.8 (4.5)§	22.9 (5.3)	25.9 (6.2)§	28.8 (6.8)§
Systolic pressure (mm Hg)	122.4 (12.5)	126.2 (14.4)‡	129.1 (16.5)†	121.6 (17.6)	120.0 (17.3)	125.0 (20.6)†
Diastolic pressure (mm Hg)	77.3 (9.5)	78.5 (10.1)	79.6 (10.8)	81.8 (12.7)	79.2 (10.9)*	82.8 (12.3)‡
Pulse rate (beats per minute)	65.8 (9.2)	65.8 (9.2)	67.0 (9.5)	69.0 (12.2)	69.4 (10.9)	69.6 (11.6)
Serum total cholesterol (mmol/L)	5.00 (0.98)	5.06 (1.03)	5.36 (1.12)§	4.50 (1.23)	5.33 (1.53)§	5.54 (1.43)
Serum HDL cholesterol (mmol/L)	1.45 (0.41)	1.45 (0.40)	1.39 (0.37)*	1.53 (0.60)	1.44 (0.52)	1.34 (0.44)*
HDL-to-total cholesterol ratio	0.30 (0.09)	0.30 (0.09)	0.27 (0.09)§	0.35 (0.12)	0.28 (0.10)§	0.25 (0.09)‡
Plasma glucose (mmol/L)	4.89 (1.06)	5.05 (1.36)	5.11 (1.20)	5.09 (0.96)	5.37 (1.28)†	5.57 (1.08)*
Geometric mean (IQR)						
γ-glutamyltransferase (units/L)	15.8 (11.0–20.9)	17.0 (12.0–22.9)	20.0 (12.9–28.8)§	50.3 (25.7–83.2)	41.7 (22.4–64.6)*	41.7 (22.4–57.5)
dp-ucMGP (μg/L)	1.97 (1.72–2.60)	3.85 (3.43–4.22)§	6.69 (5.31–7.51)§	1.88 (1.27–3.51)	5.69 (5.19–6.39)§	9.02 (7.86–10.1)§
t-ucMGP (mg/L)	45.2 (37.6–56.9)	43.2 (35.9–58.3)	47.4 (38.5–59.6)*	…	…	…

Abbreviations: dp-ucMGP, desphospho-uncarboxylated matrix Gla protein (MGP); t-ucMGP, total uncarboxylated MGP; HDL, high-density lipoprotein; IQR, interquartile range. To convert dp-ucMGP from μg/L into pmol/L and t-ucMGP from mg/L into nmol/L, multiply by 94.299. Hypertension was a blood pressure of ≥140 mmHg systolic or ≥90 mmHg diastolic, or use of antihypertensive drugs. Diabetes mellitus was a fasting plasma glucose ≥7.0 mmol/L (126 mg/dL) or use of antidiabetic agents. Significance of the difference with the adjacent lower third within each cohort: * *P*≤0.05; † *P*≤0.01; ‡ *P*≤0.001; and § *P*≤0.0001. An asterisk indicates data not available.

Table S2

Characteristics of South African participants by ethnicity and thirds of the dp-ucMGP distribution.

Characteristic	Whites			Blacks		
	Category of dp-ucMGP			Category of dp-ucMGP		
Limits, (μg/L)	<5.73	5.73–8.04	≥8.04	<2.76	2.76–5.87	≥5.87
N° participants (%)						
N° in category	120	122	120	117	117	118
Women	74 (61.7)	65 (53.3)	66 (55.0)	38 (32.5)	66 (56.4)‡	80 (67.8)
Smokers	22 (18.3)	16 (13.1)	15 (12.5)	87 (74.4)	73 (62.4)*	51 (43.2)†
Drinking alcohol	81 (67.5)	85 (69.7)	72 (60.0)	84 (71.8)	82 (70.1)	74 (62.7)
Hypertension	13 (10.8)	16 (13.1)	19 (15.8)	41 (35.0)	32 (27.4)	47 (39.8)*
Antihypertensive treatment	21 (17.5)	21 (17.2)	29 (24.2)	0 (0)	0 (0)	0 (0)
Diabetes mellitus	4 (3.3)	12 (9.8)*	11 (9.2)	1 (0.8)	3 (2.6)	6 (5.1)
HIV positive	1 (0.8)	0 (0)	0 (0)	31 (26.5)	40 (34.2)	33 (28.0)
Mean (SD)						
Age (years)	36.8 (12.5)	40.7 (13.1)*	43.6 (12.4)	39.4 (11.4)	38.6 (10.3)	44.7 (12.0)§
Body mass index (kg/m^2)	25.9 (5.2)	27.7 (6.0)*	29.6 (5.6)†	21.6 (5.0)	23.9 (6.0)†	26.4 (8.3)†
Systolic pressure (mm Hg)	116.2 (14.6)	119.7 (17.7)	121.5 (14.9)	124.0 (18.5)	123.5 (18.2)	128.7 (24.4)*
Diastolic pressure (mm Hg)	76.0 (9.2)	78.2 (10.1)	80.1 (9.8)	84.0 (13.9)	83.4 (11.6)	86.4 (14.0)
Pulse rate (beats per minute)	67.6 (9.0)	67.0 (8.9)	67.8 (9.6)	68.4 (12.9)	71.1 (13.0)	74.2 (13.7)
Serum total cholesterol (mmol/L)	5.84 (1.29)	5.90 (1.39)	5.84 (1.58)	4.29 (1.10)	4.22 (1.05)	4.57 (1.08)*
Serum HDL cholesterol (mmol/L)	1.46 (0.41)	1.41 (0.42)	1.28 (0.36)*	1.62 (0.70)	1.45 (0.62)*	1.40 (0.52)
HDL-to-total cholesterol ratio	0.26 (0.08)	0.25 (0.08)	0.23 (0.08)*	0.38 (0.13)	0.35 (0.12)*	0.31 (0.10)*
Plasma glucose (mmol/L)	5.39 (1.04)	5.66 (1.56)	5.57 (0.83)	5.02 (0.65)	5.02 (0.94)	5.35 (1.34)*
Geometric mean (IQR)						
γ-glutamyltransferase (units/L)	27.5 (17.8–38.0)	29.5 (20.0–38.9)	34.7 (21.9–50.1)*	63.1 (30.9–91.2)	66.1 (33.1–125.9)	69.2 (27.5–166.0)
dp-ucMGP (μg/L)	4.12 (3.76–5.19)	6.85 (6.24–7.68)§	9.90 (8.82–10.6)§	1.06 (0.57–2.07)	4.03 (3.43–4.85)§	7.86 (6.54–8.82)§

Abbreviations: dp-ucMGP, desphospho-uncarboxylated matrix Gla protein (MGP); t-ucMGP, total uncarboxylated MGP; HDL, high-density lipoprotein; IQR, interquartile range. To convert dp-ucMGP from μg/L into pmol/L and t-ucMGP from mg/L into nmol/L, multiply by 94.299. Hypertension was a blood pressure of ≥140 mmHg systolic or ≥90 mmHg diastolic, or use of antihypertensive drugs. Diabetes mellitus was a fasting plasma glucose ≥7.0 mmol/L (126 mg/dL) or use of antidiabetic agents. Significance of the difference with the adjacent lower third: * P≤0.05; † P≤0.01; ‡ P≤0.001; and § P≤0.0001.

Table S3

Characteristics of Flemish participants by thirds of the t-ucMGP distribution.

**Characteristic**		**Categories of t-ucMGP**		***P***
Limits, (mg/L)	<40.2	40.2–53.6	≥53.6	
Number of participants (%)				
All patients in category	389	388	389	
Women	219 (56.3)	190 (49.0)*	189 (48.6)	0.053
Smokers	88 (22.6)	77 (19.8)	91 (23.4)	0.45
Drinking alcohol	229 (58.9)	252 (65.0)	239 (61.4)	0.22
Hypertension	105 (27.0)	113 (29.1)	119 (30.6)	0.54
Antihypertensive treatment	46 (11.8)	69 (17.8)*	53 (13.6)	0.053
Diabetes mellitus	19 (4.9)	9 (2.3)	25 (6.4)†	0.021
Previous cardiovascular disease	9 (2.3)	15 (3.9)	11 (2.8)	0.43
Mean (SD) of characteristic				
Age (years)	38.9 (15.8)	38.4 (16.7)	37.2 (14.9)	0.34
Body mass index (kg/m^2)	25.0 (4.1)	25.2 (4.1)	26.1 (4.6)†	0.0006
Systolic pressure (mm Hg)	124.6 (14.4)	126.6 (15.4)	126.6 (14.5)	0.11
Diastolic pressure (mm Hg)	78.1 (10.1)	78.1 (10.4)	79.2 (10.0)	0.18
Pulse rate (beats per minute)	65.6 (9.4)	65.9 (9.7)	67.1 (8.8)	0.064
Serum total cholesterol (mmol/L)	4.98 (1.03)	5.05 (1.01)	5.39 (1.08)§	<0.0001
Serum HDL cholesterol (mmol/L)	1.48 (0.40)	1.43 (0.39)	1.38 (0.38)	0.0033
HDL-to-total cholesterol ratio	0.31 (0.10)	0.29 (0.09)*	0.27 (0.09)§	<0.0001
Plasma glucose (mmol/L)	4.96 (1.21)	4.97 (1.04)	5.11 (1.36)	0.16
Geometric mean (IQR) of characteristic				
γ-glutamyltransferase (units/L)	16.2 (11.0–20.9)	16.6 (11.0–21.9)	20.4 (12.9–28.2)§	<0.0001
dp-ucMGP (µg/L)	3.68 (2.60–5.08)	3.64 (2.60–5.44)	3.76 (2.66–5.63)	0.67
t-ucMGP (mg/L)	29.9 (26.6–37.6)	46.3 (43.2–49.6)§	65.4 (58.3–71.7)§	<0.0001

Abbreviations: dp-ucMGP, desphospho-uncarboxylated matrix Gla protein (MGP); t-ucMGP, total uncarboxylated MGP; HDL, high-density lipoprotein; IQR, interquartile range. To convert dp-ucMGP from µg/L into pmol/L and t-ucMGP from mg/L into nmol/L, multiply by 94.299. Hypertension was a blood pressure of ≥140 mmHg systolic, or ≥90 mmHg diastolic, or use of antihypertensive drugs. Diabetes mellitus was a fasting plasma glucose ≥7.0 mmol/L (126 mg/dL) or use of antidiabetic agents. p values denote the significance of the difference in prevalence or mean across thirds of the distribution of t-ucMGP. Significance of the difference with the adjacent lower third: * *P*≤0.05; † *P*≤0.01; ‡ *P*≤0.001; and § *P*≤0.0001.

Table S4

Renal function in Flemish participants by thirds of the t-ucMGP distribution.

**Characteristic**	**Category of t-ucMGP**			***P***
Limits, (mg/L)	<40.2	40.2–53.6	≥53.6	
Number of participants (%)				
All patient in category	389	388	389	
Microalbuminuria	21 (5.4)	19 (4.9)	13 (3.3)	0.36
Stage of chronic kidney disease				
1	174 (44.7)	180 (46.4)	189 (48.6)	
2	198 (50.9)	185 (47.7)	180 (46.3)	0.65
3	17 (4.4)	23 (5.9)	20 (5.1)	
Mean (SD) of characteristic				
Serum creatinine (μmol/L)	84.9 (14.9)	86.3 (14.9)	85.6 (14.7)	0.45
eGFR (mL/min/1.73 m^2)	88.7 (19.8)	89.1 (20.0)	90.7 (19.4)	0.32
Geometric mean (IQR) of characteristic				
Urinary ACR (mg/mmol)	0.82 (0.46–1.42)	0.80 (0.43–1.38)	0.76 (0.42–1.32)	0.38
24-h microalbuminuria (mg)	8.6 (4.9–15.0)	8.5 (4.9–15.0)	8.0 (4.6–15.0)	0.41

Abbreviations: t-ucMGP, total uncarboxylated matrix Gla protein (MGP); eGFR, estimated glomerular filtration rate according to the Chronic Kidney Disease Epidemiology Collaboration (CKD-EPI) equation; IQR, interquartile range; ACR, urinary albumin-to-creatinine ratio. Microalbuminuria was a 24-h urinary albumin-to-creatinine of ≥3.5 mg/mmol for women and ≥2.5 mg/mmol for men. Chronic kidney disease (CKD) was staged according to the National Kidney Foundation (KDOQI) guideline as eGFR ≥90, 60–89, 30–59 mL/min/1.73m^2. *P* values denote the significance of the differences in prevalence or mean across thirds of the distribution of t-ucMGP.

Table S5

Unadjusted associations of renal function with desphospho-uncarboxylated matrix Gla protein.

Participants<br>Model	eGFR (mL/min/1.73 m^2)		Chronic kidney disease	
	Association size (95% CI)	*P*	Odds ratio (95% CI)	*P*
White Flemish	–3.57 (–4.26 to –2.22)	<0.0001	1.35 (1.17 to 1.55)	<0.0001
White South Africans	–2.01 (–3.42 to 1.41)	0.25	1.06 (0.73 to 1.55)	0.74
Black South Africans	–4.02 (–5.62 to –2.42)	<0.0001	1.44 (1.05 to 1.97)	0.024
All Whites	–0.58 (–1.77 to 0.61)	0.34	1.01 (0.90 to 1.13)	0.87
All South Africans	–6.98 (–8.37 to –5.60)	<0.0001	1.58 (1.27 to 1.95)	<0.0001
All Participants	–3.60 (–4.67 to –2.53)	<0.0001	1.15 (1.04 to 1.26)	0.0044

Association sizes and odds ratios express the change in the dependent variable associated with a doubling of desphospho-uncarboxylated matrix Gla protein (dp-ucMGP).

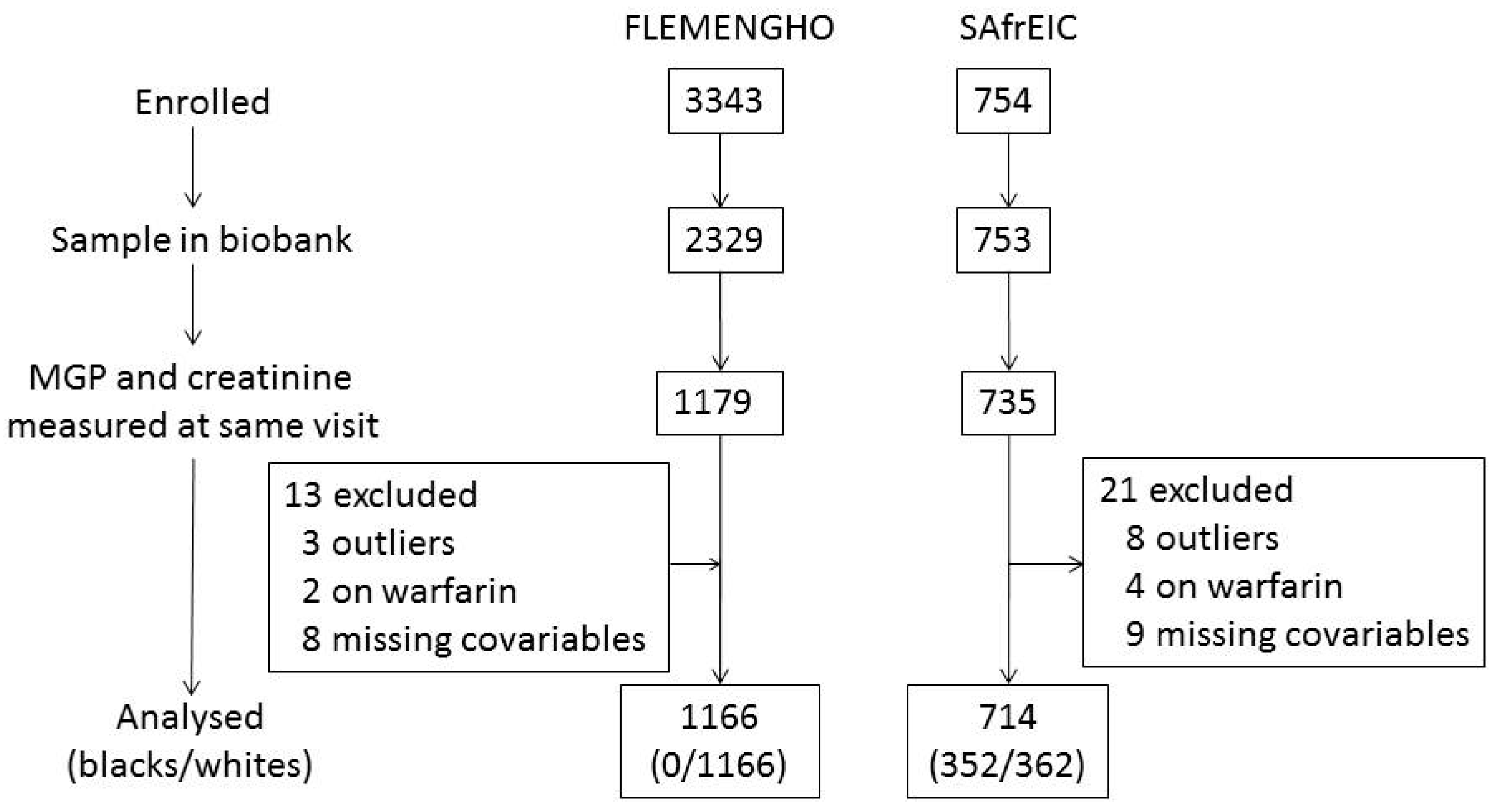

Fig. S1. Flow chart of participants.

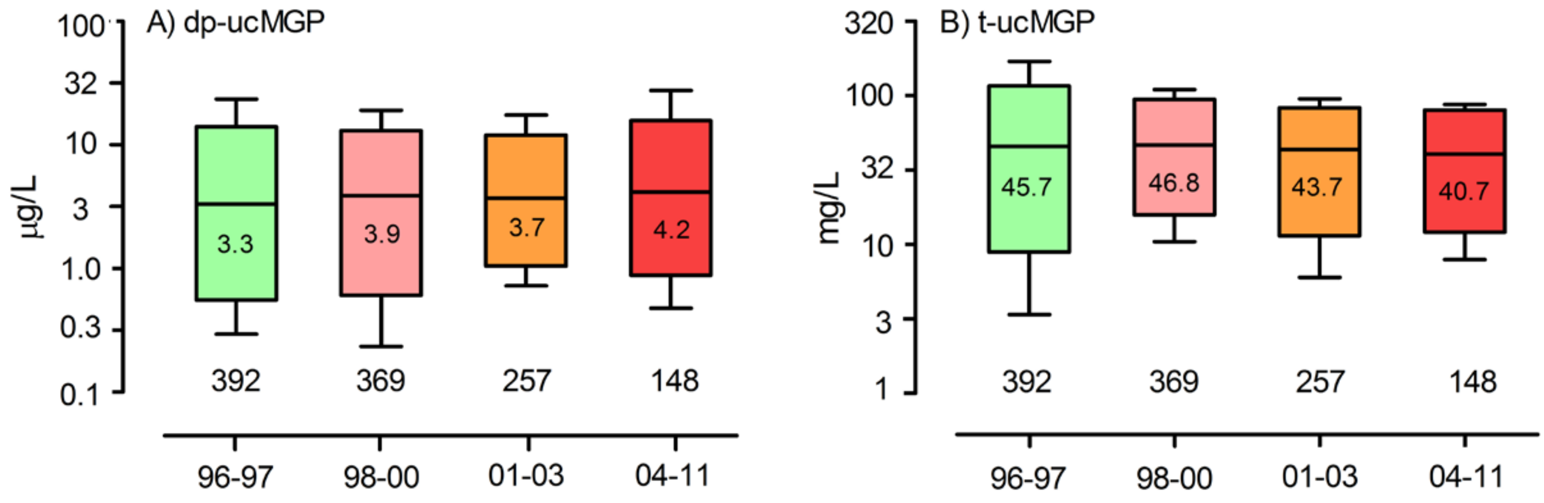

Fig. S2. Boxplots of the distributions of matrix Gla protein by date of blood sampling in FLEMENGHO participants.

Box plots represent the median, interquartile range, and 5th to 95th percentile interval for desphospho-uncarboxylated matrix Gla protein (dp-ucMGP; panel A) and for total uncarboxylated matrix Gla protein (t-ucMGP; panel B). Intervals encompass the years 1996-1997 (n=392), 1998-2000 (n=369), 2001-2003 (n=257), and 2004-2011 (n=148).

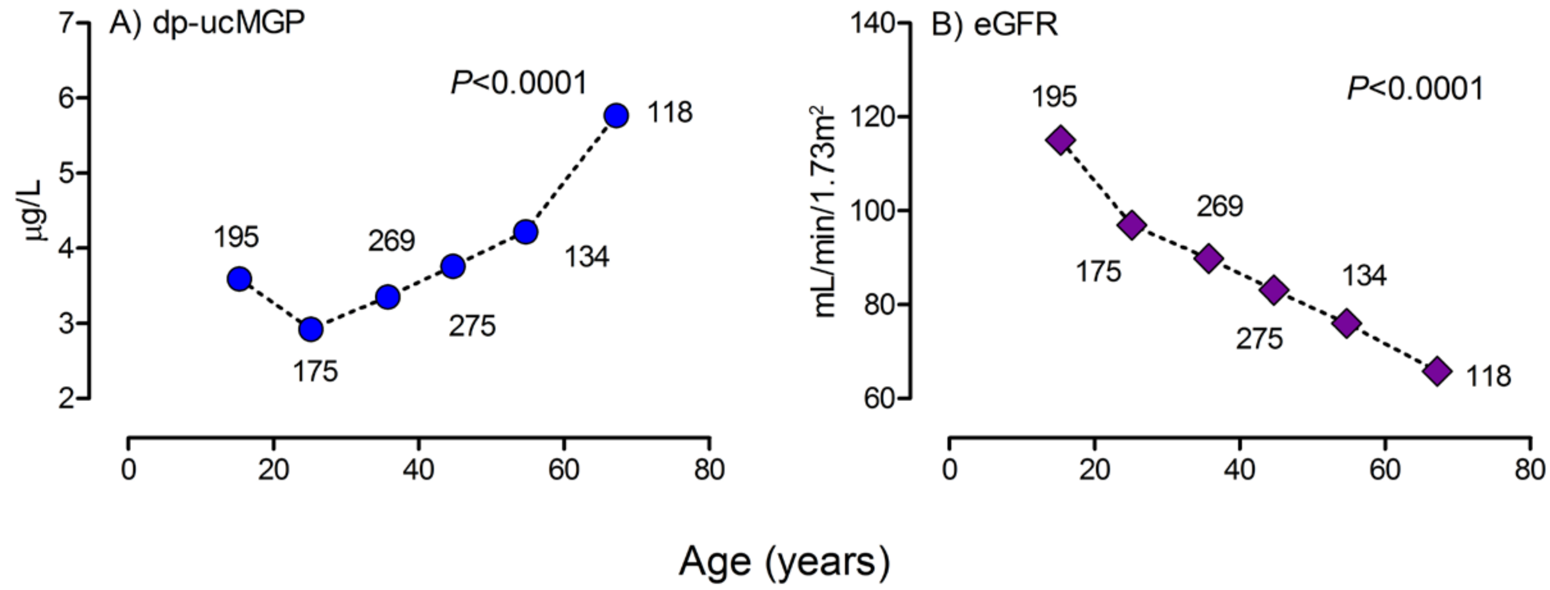

Fig. S3. Age dependency of circulating matrix Gla protein and estimated glomerular filtration rate in 1166 FLEMENGHO participants. dp- ucMGP (panel A) refers to desphospho-uncarboxylated matrix Gla protein. eGFR (panel B) is the glomerular filtration rate derived from serum creatinine according to the Chronic Kidney Disease Epidemiology Collaboration equation. Plotted values are geometric means for dp- ucMGP and arithmetic means for eGFR. The number of participants contributing to the statistic in each age group is given alongside the plotted values. *P* values denote the significance of the differences across the age groups.

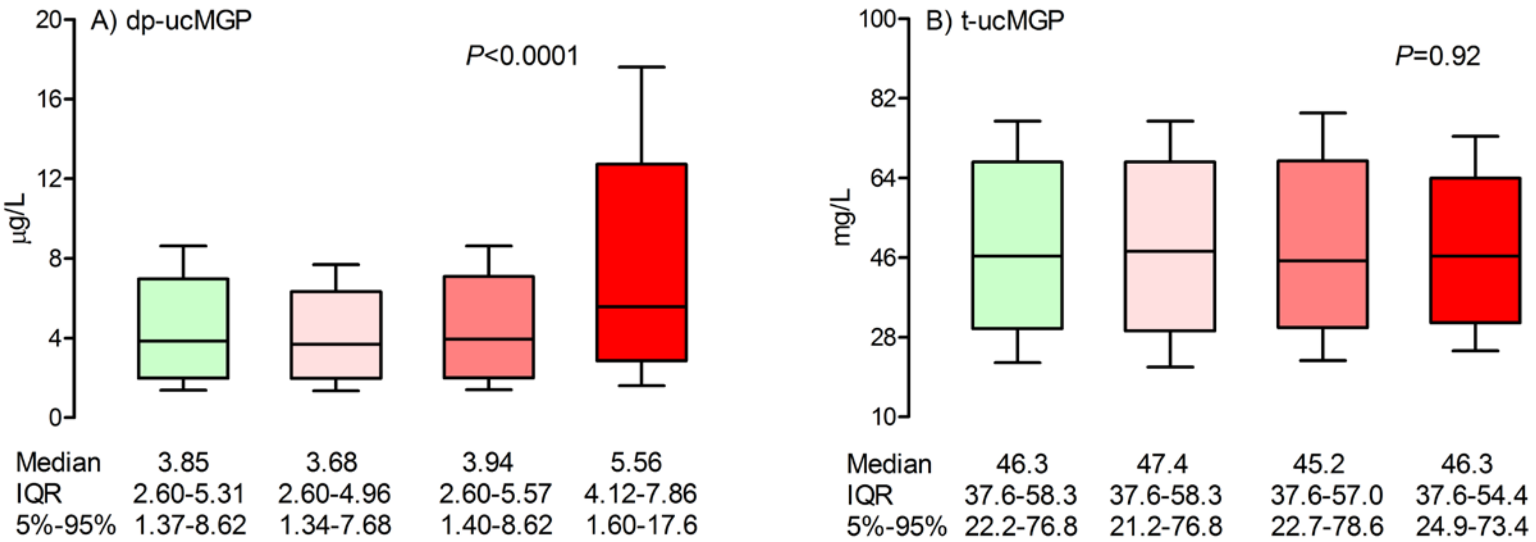

Fig. S4. Association between circulating matrix Gla protein levels and stage of chronic kidney disease in 1166 FLEMENGHO participants. Box plots represent the median, interquartile range, and 5th to 95th percentile interval for desphospho-uncarboxylated matrix Gla protein (dp-ucMGP; panel A) and for total uncarboxylated matrix Gla protein (t-ucMGP; panel B) in all participants (n=1166) and in participants with stages 1 (n=543 [46.6%]), 2 (n=563 [48.3%]), and 3 (n=60 [5.1%]) of chronic kidney disease according to the National Kidney Foundation (KDOQI) guideline (estimated glomerular filtration rate, ≥90, 60–89, 30–59 mL/min/1.73 m^2, respectively. *P* values are for the association between stage of chronic kidney disease and the matrix Gla protein levels.

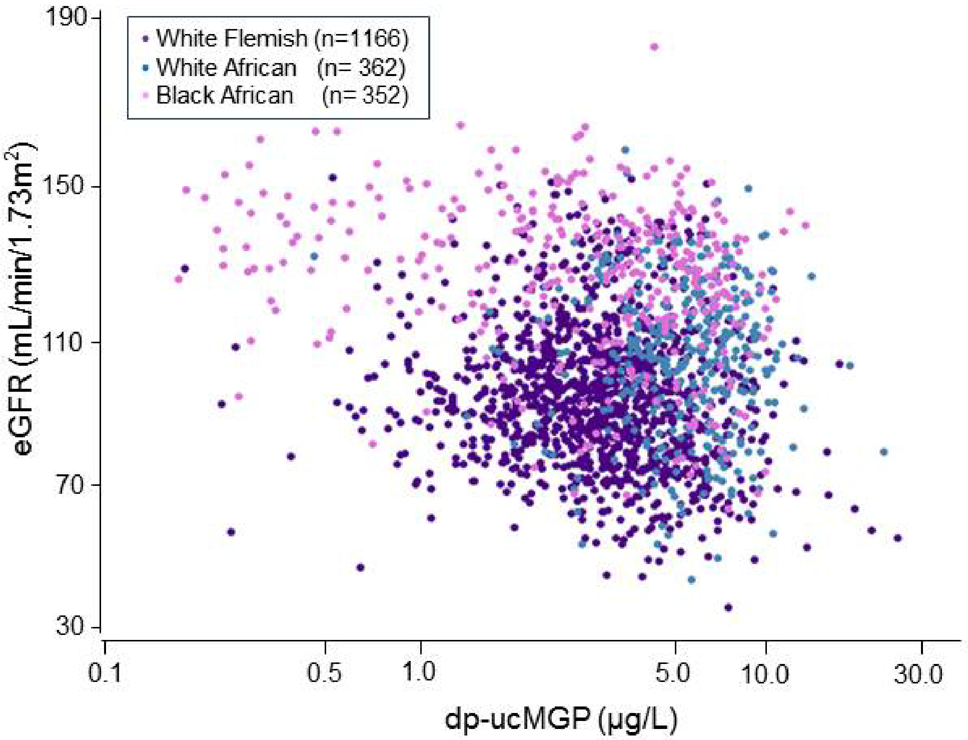

Fig. S5. Association between estimated glomerular filtration rate and desphospho-uncarboxylated matrix Gla protein in 1880 participants.

The plot includes black Africans (n=352; r=-0.25; *P*<0.0001), white Flemish (n=1166 r=-0.15; *P*<0.0001) and white Africans (n=362; r=-0.061; *P*=0.25).
